# Values of Alpha 1 Microglobulin Does Not Differ between Individuals with and without Family History of Balkan Endemic Nephropathy

**DOI:** 10.1155/2014/284293

**Published:** 2014-01-19

**Authors:** Mirna Aleckovic-Halilovic, Enisa Mesic, Senaid Trnacevic, Emir Hodzic, Vildana Habul, Mirza Atic, Maida Dugonjic, Evlijana Hasanovic

**Affiliations:** Department of Nephrology, Clinic for Internal Diseases, Dialysis and Kidney Transplantation, University Clinical Centre Tuzla, Trnovac bb, 75000 Tuzla, Bosnia and Herzegovina

## Abstract

*Aim*. The aim of this study was to compare urinary alpha 1 microglobulin (A1MG) in healthy individuals with and without family burden for Balkan endemic nephropathy (BEN) in an endemic village. *Methods*. Otherwise healthy inhabitants with microalbuminuria or proteinuria were divided into two groups: with (*n* = 24) and without (*n* = 32) family BEN burden and screened for urinary A1MG and A1MG/urine creatinine ratio. *Results*. Average value of urinary A1MG was 10.35 ± 7.01 mg/L in group with and 10.79 ± 8.27 mg/L in group without family history for BEN (NS, *P* = 0.87). A1MG was higher than 10 mg/L in eight (33.33%) inhabitants with family history and in 12 (37.5%) without (NS, *P* = 0.187). Average values of urinary A1MG/creatinine ratio were 1.30 ± 1.59 and 0.94 ± 0.78 in group with and group without family BEN history (NS, *P* = 0.39, resp.). Elevated values of this ratio were found in 13 (54.17%) inhabitants with and 14 (43.75%) without family history for BEN (NS, *P* = 0.415). *Conclusion*. We did not find statistically significant difference in the examined markers between healthy individuals with and without family burden for BEN. We concluded that these markers are not predictive of risk for BEN.

## 1. Introduction

Balkan endemic nephropathy (BEN) is chronic irreversible tubulointerstitial nephritis that is diagnosed in agrarian regions of the Balkan (Bosnia and Herzegovina, Bulgaria, Croatia, Romania, and Serbia). BEN has never been documented in children and adolescents, has insidious onset, and is usually diagnosed in the fifth decade with terminal renal failure developing in sixth and seventh decade of life. The disease is also characterized by frequent occurrence of tumours of the renal pelvis and urethers. Majority of endemic villages are located at the alluvial valleys of Danube and its tributary rivers. There have been no significant changes in the geographic distribution of BEN since its first descriptions in 1950s [1, 2]. During the past half century, numerous dilemmas and conflicting opinions regarding BEN aetiology have been proposed and the theory that BEN is caused by chronic poisoning with aristolochic acid ingested by food in people with genetic predisposition has gained credence [3]. Based on these reports, BEN is proposed to be categorized as a toxic tubulointerstitial nephropathy, with clinical picture and disease progression not different from other tubulointerstitial nephropathies, but with BEN having an insidious and gradual progression to end stage renal disease. Despite this encouraging leads to the explanation of its aetiology, this endemic kidney disease remains a mystery since its focal and stable distribution for more than 50 years remains unexplained [2]. It is well known that tubular proteinuria, intermittent in the beginning and persistent later on, is one of the early signs of BEN. There are several low molecular weight protein markers suggesting tubular damage. In accordance with our former studies [4], recent studies of cadmium nephropathy in Japan [5] and in BEN [6], it seemed that alpha 1 microglobulin (A1MG) could be good candidate biomarker of tubular damage in BEN, but there are many conflicting results regarding its superiority to beta 2 microglobulin (B2MG). The aim of this study was to evaluate urinary excretion of A1MG in healthy individuals with and without epidemiological and family risk for BEN in one of endemic villages in our country.

## 2. Methods

Members of Society of Nephrology, Dialysis and Transplantation in Bosnia and Herzegovina have organised screening in one village of Bosanska Posavina, Domaljevac, a known endemic village located near Sava River, between the towns Samac and Odzak. Inhabitants received a general call for health checkup that included history, physical exam, urine dipstick test (Combur-Test Urine test strips, Cobas, Roche) and urine test for microalbuminuria in those with negative proteinuria (Micral-Test, ACCU-CHEK, Roche). Screening was done by nephrologists, medical students, local physicians, and nurses. The checkup completed in 387 inhabitants (around 10% of total village population), between 9 and 91 years old. Over 60% of examinees were women. We divided examinees into two groups: examinees with family burden for BEN and examinees without family burden for BEN. 149 (38.5%) Domaljevac inhabitants reported positive family history of BEN. Few months after the initial screening, we invited subjects with proteinuria or microalbuminuria for additional testing in accordance with the project of early detection of chronic renal disease in high risk groups in Bosnia and Herzegovina [7]. In Domaljevac, 101 inhabitant came for additional screening, out of whom 43 (42,57%) had positive family history of BEN, while 58 (57.43%) did not. All individuals were interviewed and examined again (including body weight and height) and BMI was calculated. Repeated measurements of blood pressure over 140/90 mmHg were assumed hypertension, as well as earlier diagnosis of hypertension or usage of antihypertensives.

Urine was examined by dipstick method, for determination of microalbuminuria, creatinine in urine (by kinetic method with alkali picrate in an Architect ci/8200-Abbott Diagnostics, Wiesbaden, Germany), and urinary A1MG (by an immunonephelometric method using Behring Nephelometer II (BN II)-Dade Behring, Marburg, Germany), and A1MG/creatinine ratio was calculated. The cut-off value for A1MG for BEN screening purposes was 10 mg/L, according to the recommendations of International Workshop on Screening, Diagnosis, Classification and Treatment of Endemic (Balkan) Nephropathy [8], taking into consideration variations due to sex, age, and volume of diuresis [9]. Cut-off value for urinary A1MG (mg) to creatinine (mmol) ratio was 0.7 mg/mmol [10].

Using standard biochemical methods, blood levels of creatinine, fasting glucose, cholesterol HDL, LDL, triglycerides, and hemoglobin were determined, with values expressed in SI units. Glomerular filtration rate was estimated (eGFR) in all individuals, using the MDRD formula. Ultrasound examination of kidneys was done by two experienced nephrologists (Logic 3 General Electric device with 3,75 MHz probe), with measurement of length, width, and parenchyma thickness. Average length was calculated by adding length of both kidneys and dividing it by two. Values lower than 100 mm were assumed to be reduced kidney length [11].

Individuals with known or newly diagnosed diabetes were excluded from further analyses (five with positive BEN family history and seven without). Since it is known that hypertension may alter excretion of A1MG [12], we also excluded individuals with newly diagnosed or treated hypertension (14 with and 19 without family history of BEN). After these exclusions, the final data processing included 24 inhabitants with positive family history of BEN and 32 without.

### 2.1. Statistical Analysis

Besides descriptive statistics, we used inferential statistics for data analysis. D'Agostino-Pearson test showed that urinary A1MG and A1MG/creatinine ratio did not have normal distribution, while all other variables were normally distributed. Statistical significance of difference in numerical variables between the groups were tested using Mann-Whitney Test (small samples), while differences for nominal variables were tested using Fisher's exact test (small samples).

## 3. Results

Demographic structure of the examined population was similar in both groups (Table 1) and we did not find significant differences in frequency of proteinuria or albuminuria between the groups (Table 2). Average blood pressure was normal in both groups (median 120/80 mmHg). BMI median was 24.95 for examinees with family history of BEN and 25.7 for those without family burden, without statistically significant difference (Mann-Whitney Test *P* = 0.37). Hematuria was found in eight examinees (33.33%) in group with family burden for BEN and in five examinees (15.63%) in group without family burden for BEN (statistically insignificant-Fisher's exact test *P* = 0.200). Average values of creatinine, eGRF and hemoglobin in both groups were in normal ranges.

Table 2 depicts differences between two examined groups in frequency and average values of albuminuria and average values of A1MG, kidney length, and cortex width that were not statistically significant. A1MG was elevated (higher than 10 mg/L) in eight (33.33%) inhabitants with family burden and 12 (37.5%) examinees without family burden for BEN (Fisher's exact test, *P* = 0.187).

Average values of urinary A1MG/creatinine ratio were 1.30 ± 1.59 (median 0.82) and 0.94 ± 0.78 (median 0.68) in group with and group without family BEN burden, respectively, without statistically significant difference (Mann-Whitney Test, *P* = 0.39). Elevated values of this ratio in urine (higher than 0.7) were found in 13 (54.17%) inhabitants with and 14 (43.75%) inhabitants without family burden for BEN (Fisher's exact test, *P* = 0.415).

We also did not find statistically significant difference in frequency of reduced kidney length (less than 100 mm) between these groups.

## 4. Discussion

Diagnosis of BEN in early, asymptomatic stage is challenging. Even when decline in renal function exists, the symptoms and signs of the disease are not specific. Renal biopsy is performed in a very limited number of patients with BEN suspicion and BEN is still diagnosed if agreed diagnostic criteria are met and after excluding other possible renal diseases.

Basic criteria for diagnosis of BEN defined by Danilovic [13] were used until 2008, when replaced by criteria agreed upon during the international meeting on diagnostic criteria for BEN, and issued as a “Consensus Statement” [8]. It was agreed that, above all, in diagnosing, defining, and classification of BEN, internationally accepted criteria for diagnosis and classification of chronic kidney diseases (CKD) should be used [14].

This Consensus Statement [8] suggests classification of a subject as a BEN case if the following criteria are met: increased tubular proteinuria (A1MG), positive history for previous renal disease or carcinoma of urothelium, positive family history, or presence of disease in the household. Basic screening for BEN assumes getting answers for the following questions: whether the subject lives in an endemic village for more than 20 years, whether the subject lives in the affected household for more than 15 years, is there a proximal tubular proteinuria (A1MG), decreased renal function, urothelial carcinoma, and whether the subject is treated for any other disease that could cause chronic renal disease.

During all these years, different criteria were suggested or added to the accepted diagnostic criteria for BEN, in order to improve the diagnosis of BEN in its early stages. Among others, there were decreased kidney volume and shorter longitudinal diameter of the kidney found on ultrasonography. Anemia, more profound than expected for the given stage of CKD, was considered for a long time as one of the criteria for diagnosing early BEN [15]. However, in the recent times, its role in early diagnosis has been questioned. Djukanović et al. compared epidemiological, clinical, and functional markers in BEN and concluded that proteinuria, urine concentration of A1MG, and kidney length were significant predictors of BEN [11]. Overall, early diagnosis of BEN could not be made without positive family history for BEN, without data on the duration of residence in an endemic region, more accurately household, and without proof of tubular proteinuria. Nearly four decades ago, a respected epidemiologist from Bosnia and Herzegovina, Jacob Gaon, in 1976, wrote that BEN, just like other diseases, results from the action of multiple causes related to an unknown agent, environmental factors, and host-Gordon's triad [16].

In affected households, both genetically related and non-related family members are at risk, supporting the argument that familial aggregation is more important than heredity [17]. However, statistically significant direct associations were reported between the incidence of BEN and all three modes of paternal history of BEN (PHB), indicating that a major part of the effect of PHB on the onset of BEN was not mediated by clinical markers [18]. Data from Bulgaria [19] suggests that kidney function and size in the offspring of BEN patients are reduced before manifestation of the disease. In another investigation on the kidney size in the offspring of parents with BEN, it was noted that reduction of kidney length was related to mother's disease and that reduction of parenchyma was significant when both mother's parents had BEN and when descendants were older than 60 years at the time of the measurement [20].

Considering above mentioned findings, we wanted to compare certain characteristics, including A1MG and the ratio, and see if they differ between apparently healthy individuals with and without family burden for BEN, all living in endemic area, in order to define those characteristics that could be part of the pathophysiological pathway of the disease, which would make them the potential early marker of the disease.

There is a scarce number of previous studies that examined kidney function in detail in the early phases of BEN. Mainly, it was proved that primary lesion in BEN affects the proximal tubule. Radonic and Radosevic in 1992 also described changes in urinary concentrating ability, occurring some time before decease in renal blood flow and GFR [21]. In Bosnia and Herzegovina, kidney function of BEN patients in early stages of disease was examined in the late 1990s. There were 59 patients with pathohistologically confirmed diagnosis. Authors concluded that average kidney volume was bellow referent borderline values in all patients, even in the group (11 patients) with average GFR of 120.65 mL/min [22]. Twenty years later, as a part of our screening in endemic village Domaljevac, estimated GFR was found in the normal ranges regardless of family history of BEN [23].

Proximal tubular dysfunction is main characteristics of BEN. Beta 2 microglobulin (B2MG) was considered as a sensitive marker of tubulopathy for many years, and it was used in screening for BEN [24]. However, because of its instability in low pH at room temperature, nephrologists were seeking for a better and more reliable marker and finally turned to A1MG. A1MG is a 26 kDa low-molecular weight protein and a member of the lipocalin protein superfamily. It is synthesized in the liver, relatively freely filtered by glomeruli and reabsorbed by renal proximal tubules cells where it is catabolized. Due to extensive tubular reabsorption, under normal conditions, very little filtered A1MG appears in the final excreted urine. Therefore, an increase in the urinary concentration of A1MG indicates proximal tubule injury and/or impaired proximal tubular function. A1MG excretion in spot urine samples is highly correlated with the 24 h excretion [25]. It is believed that a single determination of A1MG is acceptable for screening purposes, if the urine specific gravity between is 1010 and 1030 [5].

In our current study, we did not find statistically significant differences neither in median values of A1MG, nor in frequency of elevated values of A1MG between the two groups. Similarly, no statistical difference was found in urine A1MG/creatinine ratio, proteinuria, and microalbuminuria as well as in average blood pressure, hemoglobin level, and haematuria between these two groups. Arsenović et al. had similar findings in their study, with no differences in the frequency of renal function disorders (proteinuria, alpha1-microglobulinuria, urine specific gravity, osmolality, functional excretion of sodium, and tubular phosphate resorption) or anemia between individuals with and without family BEN burden [26]. These findings are not similar with result of Imamovic et al. [27] which suggested that microalbuminuria may be a useful marker of early tubular injury in individuals at risk of developing BEN.

Ultrasound kidney measurements in our study showed smaller average values, but still in the normal limits and without any differences between examinees with positive family history of BEN and examinees without family history of BEN. In Bulgaria, in the endemic region of Vratza, it was reported that the average length of kidney in the offspring of BEN patients was significantly smaller than in the other examinees [19]. These observations were similar to those reported by Djukanovic et al. that shrinkage of kidneys starts early in the life of Balkan Nephropathy patients [28].

Since in our study we did not confirm statistically significant difference in the examined markers between healthy individuals with and without family history for BEN, we considered that these markers are probably not a part of pathophysiological pathway of the disease and are not predictive of risk for BEN. Alterations in these characteristic (increased urinary excretion of A1MG, reduced kidney length and cortex width, etc.) are probably acquired nonspecific indicators of already altered tubular and renal function in affected individuals. On the other hand, B2MG, a known early marker for BEN that we did not evaluate in our patients, could be, according to some authors, more than just a marker and may play a role in the pathogenesis of the disease [29].

We suggest screening for BEN and CKD in all inhabitants of endemic villages, regardless of family BEN burden. Although inhabitants of BEN villages without family burden for BEN have low risk of developing BEN, their risk is probably higher than that in the population outside BEN areas. These individuals should be included in regular mass screening. The incidence of BEN is underestimated since ESRD represents just the tip of the iceberg. Other stages of CKD caused by BEN represent a greater medical burden. Even though BEN shifted toward older age groups, Dimitrov et al. [30] noted that the age of onset of the disease has not changed, but the age of case identification has moved to an older age, probably because the cases were not identified at an early stage, but only after they had sought medical treatment. Shift of the disease onset to the older age groups could be explained by less intense contact with the etiological agent/agents [31], as well as by more convenient availability of preventive measures for chronic kidney and cardiovascular disease.

The increased A1MG along with increased albumin excretion in clinically healthy inhabitants of endemic area, found in our and other earlier studies, are markers of early kidney injury but are also responsible for additional adverse effect on kidney function. Thus, in analogy with enhanced secondary preventive measures in diabetics with microalbuminuria, a similar approach should be undertaken in apparently healthy inhabitants of BEN endemic areas with positive proteinuria. By detecting individuals in the early stages of CKD/BEN, we can delay progression and prevent complications by more stringent control of hypertension, dyslipidemia, hyperglycemia, anemia, electrolyte disorders, and acidosis.

## 5. Conclusion

In our study, we did not find statistically significant difference in the examined markers between healthy individuals with and without family burden for BEN. We concluded that these markers are not predictive of risk for BEN.

## Figures and Tables

**Table 1:** Demographic structure of the examined population.

	Positive family history for BEN	Negative family history for BEN
Men	11 (45.83%)	13 (41.63%)*
Women	13 (54.17%)	19 (59.37%)*
Age (median)	44 yrs	42.5 yrs*

*NS (Mann-Whitney test, *P* = 0.33).

**Table 2:** Proteinuria, microalbuminuria, A1MG, and kidney size in the examined persons.

	Positive family history for BEN (*n* = 24)	Negative family history for BEN (*n* = 31)	*P *
Proteinuria (*n*)	7 (29.17%)	5 (15.63%)*	0.403
Microalbuminuria (*n*)	8 (33.33%)	13 (40.63%)*	1.0
Microalbuminuria (mg/L)	70.0 ± 67.61	52.22 ± 67.71**	0.49
Urinary A1MG (mg/L)	10.35 ± 7.01	10.79 ± 8.27**	0.87
Kidney length (mm)	104.75 ± 7.93	104.75 ± 6.61**	0.86
Cortex width (mm)	14.15 ± 2.16	14.7 ± 1.95**	0.30

*Fisher's exact test. **Mann-Whitney test.
